# Behind open doors: Patient privacy and the impact of design in primary health care, a qualitative study in Indonesia

**DOI:** 10.3389/fmed.2022.915237

**Published:** 2022-10-19

**Authors:** Agnes Bhakti Pratiwi, Retna Siwi Padmawati, Dick L. Willems

**Affiliations:** ^1^Department of Ethics, Law, and Humanities, Amsterdam University Medical Center, Faculty of Medicine, University of Amsterdam, Amsterdam, Netherlands; ^2^Department of Medical Education and Bioethics, Faculty of Medicine, Public Health and Nursing, Universitas Gadjah Mada, Yogyakarta, Indonesia; ^3^Department of Health Behavior, Environment, and Social Medicine, Faculty of Medicine, Public Health and Nursing, Universitas Gadjah Mada, Yogyakarta, Indonesia; ^4^Center for Bioethics and Medical Humanities, Faculty of Medicine, Public Health and Nursing, Universitas Gadjah Mada, Yogyakarta, Indonesia

**Keywords:** privacy, primary health care, universal health coverage, quality of health care, Indonesia

## Abstract

**Background:**

The importance and attention to patient privacy in recent decades have been directed mostly toward medical data protection in electronic means. Hence, other aspects of patients’ privacy were overlooked, particularly in the primary health care (PHC) level. In the attempt of many countries, including Indonesia, to strive toward universal healthcare provision, a strong and accessible PHC is essential. This situation may create a tension in privacy provision where patients who need to disclose secrets may opt for other facilities, such as hospitals. This study aimed to describe and discuss patients’ and doctors’ perspectives and experiences about privacy in PHC in Indonesia, particularly since the universal coverage started.

**Design and methods:**

We used in-depth interviews and observations to gather information. Inductive and thematic data analyses were conducted. We interviewed PHC users (*n* = 17), doctors (*n* = 16), other PHC staff (*n* = 7), and non-PHC users (*n* = 5) and observed the PHC activities.

**Results:**

We found that privacy is imperative for both patients and doctors. Design and conditions in PHC, including consultation room doors open, separate rooms for treatment, and patients’ symptoms asked by other staff were aspects that undermine privacy in PHC. Inadequate physical and informational privacy protection during a patient’s visit has affected the quality of care negatively in ways that impede proper anamneses and physical examination.

**Conclusion:**

Ensuring patients’ and doctors’ physical and informational privacy is essential to creating PHC as the primary source of care that responds to the privacy values of its users, but it has been overlooked. The PHC building designs and care provision guidelines should incorporate the privacy needs of patients and doctors.

## Introduction

The existence of good-quality primary health care (PHC) facilities has become an essential instrument in the global effort of countries to provide healthcare access to everyone. This effort has become a global call for political commitment and resource investment to strengthen PHC facilities for disease prevention and health promotion. However, this investment will become less meaningful if PHC does not recognize the importance of the patients’ values, including ensuring their privacy (1). The importance and attention to patient privacy in recent decades have been focused mostly toward medical information protection in digital and electronic means, including sharing of big data (2, 3). This has shaped the scholarly, media, and policymakers’ discussion and understanding concerning patient privacy toward a narrower scope that is related to electronic data, and not the privacy experiences of doctors and patients during their visit at the healthcare facility. Previous settings where privacy have been researched are usually hospitals and emergency departments, where medical conditions are often more complex and privacy issues are relatively more profound (4–6). Other aspects of patients’ privacy, especially at the PHC level, where usually patients presenting for mild symptoms and common illness have been given less attention.

Primary health care (PHC) has been recognized as a cornerstone for providing holistic, cost-effective, equitable, and sustainable care for universal access to healthcare in developing countries (2–5). PHC should provide acceptable means of care to the society and for community empowerment (7–9). To benefit from PHC, the community needs to choose PHC as a primary source of care, which prerequisites a secure feeling of privacy (10, 11). PHC in Indonesia is provided by public PHC facilities and private general practitioner practices or clinics. The public PHC is located in almost every district, generally has better facilities, and is readier to provide healthcare services than private clinics (12). There are more than 9,000 public PHC facilities across Indonesia (12). The World Bank report in 2018 indicated concern about the lack of privacy in the public PHC, especially in rural areas (12). Although not specifically about patient privacy, previous studies in Indonesia also indicated that patients were dissatisfied with their conversation privacy with a pharmacist (13). Furthermore, some private practice general practitioners were concerned about the privacy of their tuberculosis patient data for reporting-related purposes (14). Little is known about patient privacy during their visit to a PHC in the context of the Indonesian national health insurance system.

Before the national health insurance scheme era, known as *Jaminan Kesehatan Nasional* (JKN), health insurance in Indonesia was only available for a small group of people such as civil servants, police and army, formal sector workers, and a tiny proportion of the poor covered through subsidy. The PHC in Indonesia is located in every district including remote areas and islands. The main PHC users were people who are the so-called “grassroots,” of lower socioeconomic status or living in rural areas (15, 16). The people living in cities often use any other means including hospitals or private practice. There is a common prejudice, which is not always the case, that PHC is a place to obtain free healthcare services, which has a long queue, which has uncomfortable conditions, and of low quality compared with other places. PHC is not very popular among people who have the ability to afford care elsewhere, and they usually opt for out-of-pocket payment, rather than using the NHI scheme for outpatient care at other facilities, such as hospitals and private practice (17). Since the NHI started in 2014, PHC has become more important for everyone. According to the Ministry of Health (Minister of Health Regulation number 5, year 2014), PHC is expected to handle 144 different diagnoses (18). This role implies that all kinds of patients from all ages and different diseases should be first treated at this level. Within the NHI system, PHC has an important function as the “gatekeeper,” or the first contact point to reach the rest of the healthcare system. Using the NHI, unless in cases of emergency, everyone needs to go through the PHC or other primary facilities where they are registered.

Importantly, freedom of care seekers to disclose important but sensitive information to their doctor affects directly the process and outcome of care. However, in the current and increasingly complex healthcare systems, efforts to protect patient privacy have become inextricably linked to other components of the system, including policies, leadership, and culture (2, 19). Another factor, the design of PHC by itself, may affect the experience of care for its users. Previously, the concept to view hospital as a therapeutic instrument was expressed by Michel Foucault in the 18th century (20). Reflected in his work is the understanding that the design of healthcare facilities can influence the good or bad outcome of care for patients.

Traditionally, patient privacy was determined by the professionalism of doctors. Physicians’ responsibility to protect patients’ privacy and secrecy has been articulated in the Hippocratic oath, whose core values strongly shaped the medical professional ethic culture until today (21). Internationally, among other documents, the UNESCO Universal Declaration on Bioethics and Human Rights stated the importance of respecting and protecting privacy and confidentiality of personal information (22). Privacy protection in healthcare is also considered a moral obligation to maintain patient dignity. Doctors have the ethical and legal duty to protect patients’ privacy (4).

Concerns about medical privacy protection during accessing healthcare differ between cultures. In the macro-view, privacy was known to have a root in Western societies (developed countries), where the value of individual autonomy should take precedence, whereas in other parts of the world and developing countries, the culture of prioritizing group interests is pragmatically given more weight than individual interests (19, 23). At the micro-level, when seeking medical care, patients convey information about their symptoms, disease, and their lives, which include sensitive and private information to various extent. When a patient’s privacy is violated, it may cause harm to the patient and the doctor–patient relationship (24).

Without a proper guarantee and feelings of privacy and trust toward care providers, follow-up appointments and treatment will be a challenge. This particularly affects patients with vulnerable characteristics, including minority ethnic groups, low socioeconomic status, and low trust and difficulties in communicating with healthcare providers (19). Privacy issues related to stigmatized medical conditions such as HIV/AIDS or tuberculosis (TB) are of particular importance (25). In fact, PHC is often the first place where such diagnoses or suspicions involving sensitive information are performed. Therefore, this study aimed to discuss the privacy situation in PHC and how it may affect patients through the experiences of both patients and doctors. In particular, this study aimed to describe patient privacy situation in the outpatient care setting in PHC, to identify the importance of and concerns about privacy, and to identify current situations to be taken into consideration when setting improvement measures related to patients’ privacy needs.

## Methods

### Design

This was a qualitative study on patients’ and healthcare workers’ experiences involving privacy, conducted as part of a larger study on patients’ values regarding PHC. We followed an interpretative phenomenology approach (26).

### Participants and setting

The study participants consisted of patients (PHC users and non-PHC users) and PHC providers (general practitioners, dentists, management staff, and other PHC staff). We included patients who do not use PHC as their usual source of care as they may have relevant views on privacy and may prefer other healthcare facilities because of privacy concerns. We interviewed patients and doctors in PHCs affiliated with the JKN insurance scheme about patients’ privacy, as described in Table 1. In addition, we interviewed other PHC staff and patients who do not use PHC as their regular source for healthcare. Patient participants were primarily selected purposively, recruited at two PHCs (one rural and one urban location), while healthcare workers were from five PHCs. Furthermore, some participants including non-PHC users were then reached through participants who mentioned people from their circle who did not use PHC. Interviews were conducted until data saturation, which was identified by recurring themes when we continued to interview more participants.

**TABLE 1:** Number of respondents interviewed.

Respondent	N
General practitioner	13
Dentist	3
Dual role practitioner and management	4
Other PHC staff	3
Primary health care users	17
Non-PHC users	5
Total	45

Eligible participants for PHC users were patients aged 18 years and older who have visited a PHC during the period of our study. The medical practitioners were general practitioners (doctors) or dentists with more than 6-month experience working at the PHC. Participants must be able to speak either *Bahasa Indonesia* or *Javanese* (local language).

### Data collection

The study was approved by the Medical and Health Research Ethics Committee Faculty of Medicine, Public Health and Nursing, Universitas Gadjah Mada (Reference number: KE/FK/0207/EC/2021), and approval for the study was sought from the district health office and PHCs. This study was conducted in compliance with the ethical principles for research subject protection. The researchers are as follows: ABP is a clinician and has an education background in public health, RA has an education background in anthropology, RSP is a medical anthropologist, and DLW is a former general practitioner and professor in medical ethics. All have experience in conducting qualitative research. ABP and one research assistant (RA) collected data through in-depth interviews from January to September 2021. We established triangulation of the phenomena through observations about the patient flow and daily activities in the PHC. Due to the pandemic situation, the participants were given the option to be interviewed either in-person (face-to-face) or online (through a video or phone call). Interviews were conducted at times or places chosen by the participants. Prior to interviewing, the participants were given a clear explanation about the study and provided the option for either a written or verbal informed consent upon their preference. All the participants gave consent for voice recording of the interview. The interview durations were around 60 to 90 min. We took field notes during and after the interviews.

The in-depth interviews were guided by the following topics (Supplementary file 1):

• Experience around (patient) privacy in PHC.
• The importance of (patient) privacy.
• Thoughts and concerns about (patient) privacy in PHC.

Furthermore, we referred to Karro et al. (6) and Shen et al. (2) in exploring the particular dimensions of privacy during the interviews, which include room situations during consultation session, the presence of other people, possibility of other people to overhear, and data in the medical records (2, 6).

### Data analysis

First, the interviews were transcribed verbatim and coded by two people, ABP and RA. To establish inter-coder trustworthiness, the first 10 transcripts were coded independently by ABP and RA and discussed for discrepancies and interpretations during meetings. Subsequently, every fifth transcript was coded by both ABP and RA, until all transcripts were fully coded. These steps were undertaken to establish inter-coder trustworthiness (27).

While coding the first 10 interviews, the research team regularly met to discuss the coding, resolve discrepancies, and develop categories and themes. During the meetings, we wrote a log book, including date of discussion, important findings, and disagreements that were resolved. Different interpretations during the coding were brought up and resolved during the team meetings with DL and RSP. Categories and themes emerging from the analysis were developed by ABP, refined by RA, and discussed in the regular meeting for review and finalization. ABP translated the quotations in English, which were then checked for the accuracy of translation and meanings by RSP and DLW. Data were managed and coded using NVIVO software version 11. We followed the consolidated criteria for reporting qualitative research (COREQ) (Supplementary file 2).

## Results

Table 2 shows the patient respondents’ characteristics. Most respondents are at productive age. The education level varied, with 40% of the respondents had education at junior high school or lower, almost 30% had university degree, and one was at unfinished primary school level. The occupation varied, with more than half not having a regular source of income. The highest proportion of respondents, with 27%, were housewife, and more than 20% worked in the informal sector. Around 20% were formal sector workers, who were covered by the NHI scheme called *Jaminan Kesehatan Nasional* (JKN) through the wage earner scheme. One-third of respondents were covered by the JKN non-contributory scheme, with premiums paid by the government.

**TABLE 2:** Patients’ characteristics.

Respondent number	Age (years)	Sex	Education	Occupation	Reason for visit	Means of payment
1	48	F	Primary school	Laborer	The child has asthma	JKN non-contributory
2	49	F	Primary school	Laborer	Headache and nausea	JKN non-contributory
3	65	M	Primary school	Seller	Routine visit for diabetes	JKN non-contributory
4	41	F	Bachelor degree	Housewife	Referral letter	JKN wage earner
5	64	M	Senior high school	Retired	Wound care	JKN wage earner
6	65	M	Not finished primary school	Unemployed	Wound care	JKN self-enrolled
7	74	F	Junior high school	Housewife	Routine visit for hypertension	JKN self-enrolled
8	37	F	Diploma degree	Online seller	Hemorrhoids	JKN wage earner
9	40	M	Bachelor degree	Civil servant	The child has fever	JKN wage earner
10	22	F	Senior high school	Student	Dental treatment	Out-of-pocket
11	58	F	Junior high school	Housewife	Routine visit for hypertension	JKN wage earner
12	67	M	Junior high school	Retired	Routine visit for diabetes.	JKN wage earner
13	45	F	Junior high school	Entrepreneur	Sore throat	JKN non-contributory
14	32	F	Junior high school	Housewife	The child goes for dental treatment	JKN non-contributory
15	25	F	Bachelor degree	Teacher	Antenatal care	JKN self-enrolled
16	27	F	Senior high school	Housewife	Antenatal care	JKN non-contributory
17	40	M	Senior high school	Private sector employee	Routine visit for anti-retroviral treatment	JKN self-enrolled
18	34	M	Master degree	Entrepreneur	Tiredness	Out-of-pocket
19	53	F	Senior high school	Private sector employee	Low hemoglobin	Out-of-pocket
20	39	F	Senior high school	Teacher	The child has fever	JKN non-contributory
21	32	F	Senior high school	Housewife	Gastritis	JKN self-enrolled
22	26	M	Bachelor degree	Freelance	Allergy (itchiness on the skin)	JKN self-enrolled

### Theme 1: Physical building and context of the primary health care that affects patients’ privacy

#### Consultation room doors open

The common practice in many PHCs is that consultation rooms are often left open during service provision. Most consultation areas are rooms with doors, but we observed some variations, such as consultation rooms that did not have doors, have removable room partitions, and rooms with doors but inside there is more than one consultation table. Doors were only occasionally closed, before the doctor needed to perform physical examination. Generally, it is possible to see what is going on inside the rooms, and sometimes, more than one person is provided service together in the same room. Most of the participants cited they would visit PHC for mild symptoms, but even so, this situation resulted in inconvenient feelings for some patients to talk freely and openly to the doctor. Other patients expressed that they had no objection to see a doctor in this situation; however, when given the option for doors open or closed, they preferred the latter.

An unclosed door was uncomfortable not only for patients but also for the doctor. The open doors created an opportunity and/or perception that other people present may see or hear the examination. This situation made doctors sometimes feel uneasy to ask sensitive questions in order to explore more during anamneses and also to comfortably perform physical examination. This might be one of the contributing factors to the experience patients had about the anamneses that oftentimes are very short, not elaborate enough, and physical examinations that are rarely or, if performed just shortly.

“…*If I ask about sexual behavior or private matter such as eating habits*… *I feel that they (patients) hold back some information. So they will only provide ‘good’ answers or say ‘normal’ things*… *So, this is a barrier for us to diagnose” (Respondent 26, doctor).*

Related to this situation, the majority of respondents, both patients and doctors, had the same preference of the doors being closed. Some variations of the preference are relatively related to the reason that the patients cited for the PHC visit. A patient (respondent 4), who came asking for a referral letter, cited that she did not mind the doors open because she did not need a physical examination. According to her, usually consultation room doors are open when there is no physical examination. On another occasion, she underwent dental care at the PHC, and in the dental room, it is usually closed. The lower number of dental patients than general (e.g., three dental visits compared to 100 general patients in a day) might signal that the dentist has more time flexibility for caring for patients.

*Q: When you are inside the consultation room, is the door open or closed?*

*A: Open*

*Q: For yourself, what do you think about it?*

*A: Eee*… *Because I only came for referral, so it’s still comfortable for me hehe. Because I didn’t need any (physical) examination (Respondent 4, patient).*

Other patients try to ignore the uncomfortable feeling by reassuring themselves that even with open doors, no one will try to look at what is happening inside. A patient confessed about trying not to be bothered even if someone he knows sees or listens.

“*Like in this PHC, the doors are open but no one will snoop around, except my wife and child who accompany me, that is alright. Maybe for me I wouldn’t make that as problem, but this is for me. I will try not to be bothered, so it is okay” (Respondent 9, patient).*

The reasons why the doors are usually left open were expressed by doctors, which are mainly for ease of the patient flow and to save time. Other reasons were climate (hot weather) and preventing accusation or uncomfortable situations by being alone with a patient without the presence of others as witness. A doctor mentioned that there are no complaints/objections from patients about the current situation. According to some doctors, patients will not feel disturbed with the doors open, and the doctor is able to recognize the timing about when they have to close the door for some private space with the patient. A doctor, who works at a rural PHC, would close the door only when really necessary, for example, if she needs to check the patient in a private area, but for ear examination she would not close the door.

#### Availability of separate treatment rooms

In the PHC in Indonesia, there are usually several rooms available. Commonly, a separate room is allocated for treatment, dental care, maternity and child health, TB, and HIV counseling and testing. Not every PHC has all these separate rooms, some have less facilities, and others have exceptionally complete facilities including inpatient care. A treatment room separate from the consultation room is usually available, which is also commonly used to perform physical examinations. However, if the treatment room is being used, then the patient who came from the consultation room and needs physical examination has to wait, which may take longer.

*“So if we want to examine, for example the patient has a lump on parts of their body, we want to examine but in that room there is no curtain, So, we have to move first to another room*… *to the treatment room” (Respondent 29, doctor).*

Another room is also available for specific purposes, such as to examine for TB. The TB-suspected or -confirmed patient will be in this room, and unlike the usual protocol, the doctor/staff will come to care for the patient. The patient need not buy medicines at the pharmacy counter, but it will be handed over to the patient in this room. This setting is different from the usual practice, where normally, the doctor is already inside the consultation room and the patient will walk inside from the waiting area. On the one hand, a special room is meant to prevent disease transmission, and on the other hand, the separate room and different treatment that the patients receive may raise questions and curiosity from other patients at the waiting room. This can have unintended consequences for the patient, who might have to deal with the other people’s reactions, who could be a friend, neighbor, or colleague. Similarly, this situation can arise when some PHCs have separate consultation rooms designed for voluntary counseling and testing for HIV. During the COVID-19 pandemic, patients are categorized into infectious and non-infectious cases, while fear, stigma, and misinformation about COVID-19 are still common. Some patients categorized as “infectious” choose to wait outside near the gate because of worry to be presumed to have COVID-19 infection. In the PHCs, open doors were already common practice before the COVID-19 pandemic. Consequently, there were no comments on that in the interview. In line with the report in 2018 by Rajan et al. from the World Bank, they raised the concern that almost 50% of the public PHCs did not provide privacy in consultation rooms (12). It is our impression from our interviews that lack of privacy might be an issue in most public PHCs.

#### Symptoms asked by staff other than doctors

Patients’ reason to visit were likely asked several times at different stages of their visit to the PHC. Before seeing a doctor, patients have to go to the registration desk, and there they will be asked about their reason for the visit, which often means mentioning the disease or symptoms they have. Furthermore, patients will visit the nurse station or a desk to have their vital signs and sometimes weight measured. Here, the nurse will ask some questions and fill in information in the medical record. Next, patients will meet the doctor in the consultation room, and lastly, they will take the medicine at the pharmacy. At all of these different steps, there is a possibility that patients will be asked about their disease. Patients expressed that they felt uneasy with this situation. A female patient who visited because of hemorrhoids said that she would speak with a low voice when asked about it. But she tried to bear with this uncomfortable feeling because of her pain, and getting treatment was her priority concern.

Differently, for doctors, the different steps where patients’ information is being recorded are viewed as a helpful process, to sort out some details and procedural steps related to the patients that can be shifted to other staff. It is seen as a division of tasks and workload and also to save time as the nurses can already prepare necessary documents, equipment, and/or materials related to a certain patient.

*“Because in front it is actually already filtered by the nurse for the vital signs and anamneses. So, we then confirm and complete (the medical record), and just need to explore more (information) and examine*… *The reason is for example ‘this needs wound care,’ like that the nurse can already prepare. Next ‘here is a child aged less then 5 years,’ the nurse helps to fill in the form for integrated management of childhood illness*… *So, these things won’t take long when patients see the doctor. When there is something incomplete and (patients has to) come back later, this will take a long time. So, when things are sorted out before, when patients come in (the consultation room) it will be faster” (Respondent 28, doctor).*

However, a doctor (respondent 24) acknowledges that this situation potentially leads to uncomfortable feelings for the patients, in a way that they may be unwilling to answer the questions at the registration and other steps and may only be confident to reveal honest information once they meet the doctor.

*“Sometimes when patients asked (by other staff), when patients feel that this is part of their privacy, they will not answer. And then when they enter the consultation room they start to talk frankly. Sometimes I’m also surprised. When I observe patients gesture closing the door and talk with a low volume, ah… there must be something secret” (Respondent 24, doctor).*

### Theme 2: Lack of privacy affects the quality of care negatively

At the PHC, when patients sense an unsafe and hesitant feeling of their privacy, some of the following gestures have occurred among the participants:

(1) Patients will lower their voice if there is a secret;
(2) Patients will close the door when entering the consultation room; and
(3) Patients will choose to hide and not disclose openly information relevant to their medical condition.

In addition, patients may experience uneasy, uncomfortable feelings, shame, and fear of being discovered. These negative feelings may impact the patients’ trust and safe feeling when visiting the PHC, which can deter proper anamneses, diagnoses, and then a proper treatment, particularly if there are follow-up appointments needed. Another possibility is that patients simply choose not to visit the PHC.

#### Anamneses

Not only patients but also doctors felt lack of privacy in terms of being able to be overheard and seen by other people in the PHC alienate them from quality time for anamnesis, physical examination, and diagnoses. First, related to anamneses, doctors sometimes felt uneasy and worry if their conversation with patients is listened to by other people around the area. For example, the presence of another patient in the same room makes it difficult for a doctor to extract relevant information from a teenage girl who presents with abdominal pain and later tested positive with a pregnancy test. Because when she asked questions, the patient remained silent.

*“The patient becomes less open, like they have to almost whisper because it’s close with the other doctor’s table, no curtain, no divider, just directly (between two consultation tables)” (Respondent 29, doctor).*

Another unintended impact was that doctors experience getting some comments from patients who are in the waiting room because of what the patients overheard. Although the comments might be just a spontaneous and inadvertent reaction, it may impact the doctors’ feeling about their privacy.

#### Physical examination

Second, this situation can interrupt and complicate the process for the necessary physical examination. If doctors need to examine a private area, they will go to the treatment room, which is separate from the consultation room. However, this has also a risk that other people in the waiting room may become curious.

*Q: How do you perform physical examination; how do you usually do that if there are no curtains (barrier)?*

*A: Physical examination is only minimally performed, we just decide according to the symptoms (and the patient sitting on the chair), and then when it is necessary for the patient to lie down, we usually move to the treatment room” (Respondent 29, doctor).*

Nevertheless, according to some doctors, the main constraints to perform physical examination is not privacy but more because of limited time. They would maximize the anamneses and conditionally perform physical examination according to the anamneses results.

### Theme 3: Understanding the meaning and importance of privacy

#### Doctor views

Privacy is regarded important by both patients and doctors. Most doctor respondents express the importance of privacy, and some stated explicitly that patient privacy is imperative. The importance of privacy is described by doctors in terms that it should be respected, data protection should be adequately performed, the presence of other people is considered, and if involved in research, the patient’s identity should not be identifiable. Some doctors express what privacy means to them: having a room to discuss with the patient, without other people present during consultation who may see what the doctor is doing or able to hear the conversation. Regarding patient information, it meant upholding the ethical code of keeping patients’ secrets. Privacy in the consultation room gives the doctor more focus, freedom to deepen the anamneses and talk to the patient, and perform physical examination. Privacy is also framed normatively, that is, toward the do’s and do not’s; and the “yes” and “no” whether a certain situation infringes patients’ privacy or not. This can lead to a tendency to think and classify privacy situations as “black and white,” that is, as only right or wrong, which can hamper a deeper thinking when making decisions related to patients’ secrecy or privacy issues. For instance, a doctor (respondent 27) wondered whether in the same room, two patients were examined at the same time is considered to infringe patients’ privacy or not because it is a common situation that if there are many patients in the queue, sometimes, they may examine more than one patient in the same room.

Doctors’ awareness about privacy varied. Doctors try to keep patients’ privacy by keeping secret the information they received from patients. Another doctor gave an example when she saw a patient with sexually transmitted disease who came with his wife. She would ask the wife to wait outside the room to explain the diagnosis to the patient. Oftentimes, patients’ symptoms are asked by other staff at registration and nurse station, before meeting the doctor. Doctors see this as an assistance to sort out information and hasten the service provision. However, for most patients, it means that sometimes, they have to reveal sensitive information that otherwise they would only convey to the doctor.

#### Patient values

To patients, the importance of privacy is that there is a secret that other people should not know, including what is inside the medical records. The concern is toward avoiding shame and judgment if a certain medical condition might be judged unacceptable concerning the common expectation of good behavior or norms that are related to religion, culture, and tradition. The need for privacy is mainly toward neighbors, family, and people who are close to the patient. For some, the intuition about having secure feeling of their privacy also involved being able to choose the doctor of the same gender, particularly when physical examinations are involved.

*“If the doctor checks my private area, it has to be a female. If not, I will feel uncomfortable and embarrassed hehe*… *If (the male doctor) only ask something, it is okay. However, if he checks, I do not want to. When I was pregnant, coincidentally, the doctor was male, and he wanted to check there hehe*… *There was no female doctor, so I refused the examination. The doctor went outside, and then the female nurse checked.” (Respondent 8).*

Communities in Indonesia uphold the value of togetherness and maintaining harmony, which is particularly strong in villages and small cities. The community usually still maintains a close relationship and the wisdom of helping each other in the community, which is strongly present in important occasions, such as weddings and funerals, including when someone becomes sick. The common practice is to visit (sometimes together with others or many people) who is known to be sick and offer help. This has also happened during an interview, where a neighbor came by to visit the respondent and ask about his health.

#### Privacy-sensitive issues in primary health care

Many aspects of the sensitive medical information related to patients’ privacy become part of the daily routines for the doctors meetings with patients in PHC, which can cause some dilemma for doctors. Some privacy-sensitive issues that we found in PHC and would need further attention were related to child sexual abuse, teenage pregnancy, people living with HIV, TB, sexually transmitted infection, and people with mental health issues. The community associate it with taboo, shame, or norm-violation. A patient’s family routinely visits the PHC to take referral for a family member who has HIV, but the patient will not come to visit the PHC. These patients are sometimes trapped in an uncomfortable situation that they would rather avoid.

*“For certain cases they (patients) worry. There was a teenage with the parents. Usually if there are cases suspect to sexually transmitted infection, and he is with the parents, we will ask the mother to leave (the consultation room) first. So we can freely do the anamneses” (Respondent 34, doctor).*

One PHC had a comprehensive service facility which includes the treatment for antiretroviral, sexually transmitted infection, and TB. In these patients’ medical records, stickers are placed with different colors as codes. These patients can get priority for treatment, allocated to a certain day and with a separate queue. The stickers as symbols were placed in attempt to protect the patients’ disease status, which is only understood by the PHC staff. However, this can create curiosity and questions among some other patients, who then ask the PHC staff about what is going on. The idea of stickers is used to hide the information from those who might see the file.

*“Patient will not know. So, sticker with colors for codes. More than one sticker so patients will not know that this is a sign for a specific case (on the medical record)” (Respondent 44, doctor).*

### Theme 4: Perceived privacy protection in primary health care

Patients generally had feelings of trust toward the PHC that their written (medical record) data will not be misused. Some respondents felt that PHC protects their data from other parties and knew when a referral is made, disclosure of information will be necessary. Initially, patients responded quite normative when asked about privacy experiences during their visit, describing it with the words “*it is okay*” and “*good*.” Most respondents were first showing acceptance. However, we found that this is related to the cultural value of *nrimo*, which means acceptance (28). This can be interpreted in this case into gestures and attitudes of acceptance. Among our respondents, it is the gesture and attitude of accepting what the patients receive from healthcare providers, without further questioning or complaining. In our study, this *nrimo* can be related to the respondents’ characteristics of a modest person who uses the non-contributory JKN scheme or have low education. This culture of *nrimo* is added to by the view that respondents regarded the services as provided for free, where in fact the insurance premiums are covered by the government subsidy for the JKN insurance scheme.

By contrast, some respondents explicitly explained that the privacy protection they experience was not satisfactory.

*“I think it is not enough, because it means other people may know what disease we have, listen what we say. I think every time being examined, the door has to be closed. I mean if we want to explain something, other people should not know*… *So, we can explain it more comfortably” (Respondent 4, patient).*

Similarly, doctor respondents think that if they were the patient, their privacy is more protected if examined alone without other people present. However, doctors sometimes have to see many patients (sometimes up to 100 patients per day for one PHC, with service provision, for example, from 07.30–14.30), needing speed to be able to provide services to all of them. A head of a PHC explained that patients’ privacy is sufficiently protected, which can be seen from the fact that patients trust and do not hide information from the doctor. Doctors also express their need for more privacy protection so that they can feel comfortable in doing their tasks. Some doctors realized that in the PHC setting, privacy is lacking, as such when performing physical examination, their action would be moving to another room.

*A: In PHC (privacy) is actually insufficient.*

*Q: Insufficient, like what?*

*A: So, for example we examine a lump in certain part of the patients’ body, we want to examine but there is no curtain. So we have to move to another room first” (Respondent 29, doctor).*

## Discussion

This study aimed to discuss and describe the privacy situation in PHC and how it may affect patients and the quality of care through the experiences of both patients and doctors. The feeling and perceived lack of privacy were mainly related to the physical building and setting of the PHC, and also the presence of other people in different ways, present directly in the same room, or able to see or hear what is happening during the consultation or examination of patients. This situation has affected the quality of care provision, by jeopardizing the information openness from patients to doctors, and presenting challenges for doctors in obtaining relevant medical information and performing physical examination. Inadequate privacy provision was also caused by the high patients’ load, time pressure, and the known expectation of PHCs to provide services for everyone present during the opening hours of the facility, without the obligation to make appointments the days before.

### Primary health care design and privacy

The currently common situation during the service provision reflects that the issue of privacy is not becoming a concern or priority until now. A separate room can be used for physical examination, which may well indicate situations where the patient and/or doctor feels the need for more room for privacy, although not necessarily verbally expressed as a need for privacy. The other important considerations are if the other rooms are used for specific purposes, to avoid curiosity or stigma, the patients who enter a specific room should not be visible to the rest of the patients who are in the waiting area. This arrangement also applies if for some reasons, doctors would prefer the doors in the consultation room to remain open. New PHC building designs or renovations should incorporate this need of patients and doctors for privacy. This means that a privacy discussion is not anymore mainly seen as the responsibility of the doctor but also relates to the other components of the PHC systems, including the architect and designer.

The setting in the PHC in our study setting is similar to an emergency department setting, in terms of the high patient load, which creates a time pressure, sometimes involving a shortage of clinicians, and examination rooms that are usually not completely closed with the possibility of other people able to see or overhear. Drawing lessons from the setting of an emergency unit, a study by Hartigan et al. (29) performed a change in the layout and modest renovation to a maternity emergency department unit to provide more privacy to patients. In that study, the change was made by changing from using curtains as dividers into cubicles with walls. Their before and after renovation survey showed improvement in patient privacy (29). This kind of renovation could possibly be adapted in low-resource settings with limited budget allocated to renovate the setting in PHC, and the outcomes could be analyzed if this approach may improve privacy of the users.

### Sensitive issues are prevalent in primary health care

There is a dearth in recent literature about privacy in PHC. It is possible that privacy in PHC is not a big issue anymore in countries with established primary care systems. However, in the context of a developing country, our findings reveal the fact that privacy-sensitive issues are prevalent in PHC, including issues that were not realized in the beginning by the patient but then later on was discovered by the doctor. The visit of a teenage girl who presented innocently with abdominal pain and later was discovered to be pregnant by a test result was only one among other patients. This case accentuates that sensitive patients’ information still needs to be given attention in PHC and not only in settings that are commonly known to cause issues, such as hospitals and emergency departments. We also found that the physical building and the ‘doing business as usual’ situation, with consultation doors open and sometimes other people examined in the same room, has a considerable impact on patient privacy and their comfort feeling in PHC. In addition to the physical building of the PHC, privacy protection needs to be accompanied by the professionalism attitude of staff to ensure patients’ privacy experience (29). In our study, however, we found that the unsuitable attitude is mainly due to unawareness and not intentional invasion. Privacy awareness should not only occur by itself with the experience that the doctors gained over time but should be incorporated systematically in continuing medical education and appropriate trainings.

### Leaving doors open affected the quality of care

Similar to a study in Norway that found that the ability of others to listen when healthcare providers talk to a patient is among the ethical challenges in PHC (30), we found that the importance of privacy was directed toward a safe feeling from people who are socially close to the patients, such as families and neighbors, similar to other research findings (31, 32). Worries about safeguarding medical records were relatively not expressed. Furthermore, the way ethical challenges were resolved meant more for the patients, their families, and the PHC staff (30). Similar to our findings, the specific aspect of providing privacy is of importance not only to patients but also to doctors so that they are able to talk freely to patients and perform examinations without worries about their and the patients’ privacy. Furthermore, a systematic review on family planning services found that privacy and confidentiality for the patients are an important determinant affecting the quality of care (33).

### Concerns about privacy protection

A study about privacy in perinatal care in a large hospital in Turkey by Aksoy and Komurcu found out that both women and healthcare professionals feel the need to improve physical privacy. Furthermore, most of the women patients felt insecure about their data in the medical records (34). This result differs from our findings, where patients and doctors relatively trust the PHC about their medical data privacy but mainly raised concerns about physical and informational privacy during the visit. It is possible that a different nature of the hospital such as a large institution with more complex management and information technology systems, where patient data are sent and can be accessed from one section to the other make patients more concerned, rather than in a smaller unit like PHC. A study by Humayun et al. (5) found lack of physical privacy practices in outpatient care in public and private hospitals in Pakistan, with better practices in the private hospital (5). Although this was a finding in hospitals, the notion of public and private may also have an association of people’s feelings about privacy. Our results also indicate that people may opt for private providers to avoid being seen in a public facility.

### Strengths and limitations

Our study captured views on privacy from two sides, patients and doctors. This enabled us to have a richer understanding and derive meaning on the similarity or differences in the views expressed by patients with experiences from doctors, and vice versa. We interviewed patients shortly after their attendance to PHC, which minimized the risk of recall bias. Our participants were patients who were present directly in PHC, with different purposes of visiting the PHC. The views of groups that may have more specific needs of privacy were captured more from the doctors’ perspectives. For instance, we could not reach the views of people living with HIV who do not use PHC for privacy reasons since ethically, it was not possible to reach them in this research. The culture of *nrimo* or accepting may also impact our findings, in that some respondents were not expressing their actual feeling or thoughts. This was mitigated by asking respondents to tell their experiences and then asking their thoughts and feeling related to their responses. Finally, although our respondents indicated that generally, they trust and do not have concerns about their privacy in handwritten and electronic medical records, we did not explore further on this aspect as it is beyond our study objective.

## Conclusion

In conclusion, privacy should be placed as a key element in the PHC delivery. The feeling and perceived lack of privacy lead to challenges in the information flow from patients to doctors, and vice versa. In particular, it compromised patients’ and doctors’ openness to share information and ask questions. PHCs in Indonesia, and other similar contexts, where privacy regulations are not always available in place, should give attention to the physical privacy needs of patients as well as doctors for a better doctor–patient relationship and quality of care provision. The current service provision and situation in consultation rooms in PHC do not reflect adequate consideration or priority toward patients’ privacy. Constrained resources including room designs and facilities and a priority to provide services to any patient who shows up during opening hours lead to a high load to the PHC staff, which was among the factors that contribute to inadequate privacy provision to patients. Development of policies and clear context-specific guidelines is necessary to protect patients’ and doctors’ privacy and to minimize unintended harm as a possible consequence. In light of the research findings, we thus suggest several recommendations for PHCs: (1) Develop policy to have consultation rooms with doors, and closed consultation room doors during patient encounter; (2) develop designs of PHCs with barriers, assuring that people in the waiting room should not be able to observe if patients are moved to other rooms; (3) provide context-specific trainings for healthcare workers and staff at PHC to increase awareness about the importance of patients’ privacy; and (4) conduct further research or evaluations to service users, especially those with medically sensitive information, such as people living with HIV and TB, about the special services they receive that differ from other patients.

## Data availability statement

For confidentiality reasons, full transcripts are not uploaded. Raw data are available upon request.

## Ethics statement

This study was approved by the Medical and Health Research Ethics Committee Faculty of Medicine, Public Health and Nursing, Universitas Gadjah Mada. All interview respondents provided either their written or verbal informed consent to participate in this study.

## Author contributions

AP designed the study, developed the interview guide, performed the data collection and data analysis, and developed the first manuscript draft. RP gave input and approved the study design, oversaw data collection, performed analysis, confirmed language translation in quotations, and revised the manuscript. DW supervised the study, advised and approved the study design, involved in data collection and analysis, and revised the manuscript. All authors have read and approved the final version of the manuscript.

## References

[1] Pratiwi AB Padmawati RS Mulyanto J Willem DL. Patients values regarding primary health care: A systematic review of qualitative and quantitative evidence. *BMC Health Serv Res.* (Forthcoming) (2022). 10.1186/s12913-023-09394-8 PMC10131468 37098522

[2] Shen N Sequeira L Silver MP Carter-Langford A Strauss J Wiljer D. Patient privacy perspectives on health information exchange in a mental health context: qualitative study. *JMIR Ment Health.* (2019) 6:e13306. 10.2196/13306 31719029 PMC6881785

[3] Tangari G Ikram M Ijaz K Kaafar MA Berkovsky S. Mobile health and privacy: cross sectional study. *BMJ.* (2021) 373:n1248. 10.1136/bmj.n1248 34135009 PMC8207561

[4] Geiderman JM Moskop JC Derse AR. Privacy and confidentiality in emergency medicine: obligations and challenges. *Emerg Med Clin North Am.* (2006) 24:633–56. 10.1016/j.emc.2006.05.005 16877134 PMC7132767

[5] Humayun A Fatima N Naqqash S Hussain S Rasheed A Imtiaz H Patients’ perception and actual practice of informed consent, privacy and confidentiality in general medical outpatient departments of two tertiary care hospitals of Lahore. *BMC Med Ethics.* (2008) 9:14. 10.1186/1472-6939-9-14 18816413 PMC2564960

[6] Karro J Dent AW Farish S. Patient perceptions of privacy infringements in an emergency department. *Emerg Med Australas.* (2005) 17:117–23. 10.1111/j.1742-6723.2005.00702.x 15796725

[7] Bryant JH Richmond JB. Alma-Ata and Primary Health Care: An Evolving Story. In: Kris H editor. *International encyclopedia of public health.* Oxford: Academic Press (2008). p. 152–74. 10.1016/B978-012373960-5.00001-0

[8] Shields L Hartati LE. Primary Care in Indonesia. *J Child Health Care.* (2006) 10:4–8. 10.1177/13674935063818 16464929

[9] World Health Organization. *Primary Health Care on the Road to Universal Health Coverage: 2019 Global Monitoring Report.* Geneva: WHO (2019).

[10] Deshefy-Longhi T Dixon JK Olsen D Grey M. Privacy and confidentiality issues in primary care: views of advanced practice nurses and their patients. *Nurs Ethics.* (2004) 11:378–93. 10.1191/0969733004ne710oa 15253573

[11] Olsen DP Dixon JK Grey M Deshefy-Longhi T Demarest JC. Privacy concerns of patients and nurse practitioners in primary care-An APRNet study. *J Am Acad Nurse Pract.* (2005) 17:527–34. 10.1111/j.1745-7599.2005.00078.x 16293161

[12] Rajan V Patil A Pambudi E Junaedi. *Is Indonesia Ready to Serve?: An Analysis of Indonesia’s Primary Health Care Supply-Side Readiness (English).* Washington, DC: World Bank Group (2018).

[13] Kristina S Lienaningrum A Aditama H. Assessing. patient satisfaction with community pharmacy services in Yogyakarta, Indonesia. *Int J Pharm Res.* (2021) 13:4641–45. 10.31838/ijpr/2021.13.01.652

[14] Kurniawati A Padmawati RS Mahendradhata Y. Acceptability of mandatory tuberculosis notification among private practitioners in Yogyakarta, Indonesia. *BMC Res Notes.* (2019) 12:543. 10.1186/s13104-019-4581-9 31455388 PMC6712591

[15] Post TJ. *‘Puskesmas’ move up to COVID-19 front lines amid overburdened health system.* Jakarta: The Jakarta Post (2020).

[16] Wenang S Schaefers J Afdal A Gufron A Geyer S Dewanto I Availability and accessibility of primary care for the remote, rural, and poor population of Indonesia. *Front Public Health.* (2021) 9:721886. 10.3389/fpubh.2021.721886 34621720 PMC8491579

[17] Pratiwi AB Setiyaningsih H Kok MO Hoekstra T Mukti AG Pisani E. Is Indonesia achieving universal health coverage? Secondary analysis of national data on insurance coverage, health spending and service availability. *BMJ Open.* (2021) 11:e050565. 10.1136/bmjopen-2021-050565 34607864 PMC8491299

[18] Ministry of Health Republic of Indonesia. *Minister of Health Regulation Number 5 Year 2014.* Jakarta: Ministry of Health Republic of Indonesia (2014).

[19] Knopp C. *Privacy Perception in Developing Countries.* (2019). 10.13140/RG.2.2.19821.95209

[20] Foucault M Tek E Jr King WJ Elden S. The Incorporation of the Hospital into Modern Technology. In: Crampton JW editor. *Space, Knowledge and Power: Foucault and Geography.* London: Routledge (2007).

[21] Greek Medicine. *The Hippocratic Oath.* Bethesda, MD: U.S. National Library of Medicine (2002).

[22] UNESCO. *Universal Declaration on Bioethics and Human Rights.* France: UNESCO (2006). 17139812

[23] Zhang H Zhang H Zhang Z Wang Y. Patient privacy and autonomy: a comparative analysis of cases of ethical dilemmas in China and the United States. *BMC Med Ethics.* (2021) 22:8. 10.1186/s12910-021-00579-6 33531011 PMC7856764

[24] DeCew J. Privacy. In: Zalta EN editor. *The Stanford Encyclopedia of Philosophy [Internet]. Spring 2018.* Stanford, CA: Stanford University (2018).

[25] Dapaah JM Senah KA. HIV/AIDS clients, privacy and confidentiality; the case of two health centres in the Ashanti Region of Ghana. *BMC Med Ethics.* (2016) 17:41. 10.1186/s12910-016-0123-3 27422295 PMC4947355

[26] Elliott R Timulak L. Why a generic approach to descriptive-interpretive qualitative research? In: Elliott R Timulak L editors. *Essentials of descriptive-interpretive qualitative research: A generic approach.* Washington, DC: American Psychological Association (2021). 10.1037/0000224-000

[27] O’Connor C Joffe H. Intercoder reliability in qualitative research: debates and practical guidelines. *Int J Qual Methods.* (2020) 19:1–13. 10.1177/1609406919899220

[28] Erlina E. *The Healing Corridor: A Critical Phenomenology of Severe Illness, Impairment and Care in Java.* Ph.D. thesis. Australia: The Australian National University (2020).

[29] Hartigan L Cussen L Meaney S O’Donoghue K. Patients’ perception of privacy and confidentiality in the emergency department of a busy obstetric unit. *BMC Health Serv Res.* (2018) 18:978. 10.1186/s12913-018-3782-6 30563545 PMC6299575

[30] Lillemoen L Pedersen R. Ethical challenges and how to develop ethics support in primary health care. *Nurs Ethics.* (2013) 20:96–108. 10.1177/0969733012452687 22918061

[31] Edwards PV Roberts ST Chelwa N Phiri L Nyblade L Mulenga D Perspectives of adolescent girls and young women on optimizing youth-friendly HIV and sexual and reproductive health care in Zambia. *Front Glob Womens Health.* (2021) 2:723620. 10.3389/fgwh.2021.723620 34816241 PMC8594040

[32] Sheikhansari N Abraham C Denford S. Health-care professionals’ assessments of, and recommendations for, sexual-health education and service provision for young people in Tehran. *Front Public Health.* (2021) 9:634795. 10.3389/fpubh.2021.634795 34504822 PMC8421759

[33] Tessema GA Streak Gomersall J Mahmood MA Laurence CO. Factors determining quality of care in family planning services in Africa: a systematic review of mixed evidence. *PLoS One.* (2016) 11:e0165627. 10.1371/journal.pone.0165627 27812124 PMC5094662

[34] Aksoy S Komurcu N. Privacy in perinatal services: a qualitative study. *Nurs Health Sci.* (2018) 7:64–73.

