# Supplementary material for: Behind open doors: Patient privacy and the impact of design in primary health care, a qualitative study in Indonesia
Source: Front Med (Lausanne). 2022 Oct 19;9:915237. doi: 10.3389/fmed.2022.915237 (PMC9626974; doi:10.3389/fmed.2022.915237)
Supplement: Supplementary file 1 [file Table_1.DOCX]

**Supplementary File 1. Interview Guide**

**Primary Health Care (PHC) users**

What is the purpose of your visit (to the PHC)?

What is your experience with privacy during your visit to a PHC?

What do you think about your privacy during the different steps in PHC?

Explore the aspects:

-Privacy during registration, vital sign measurement, consultation, and examination.

-Presence of other people during consultation/examination

-Possibility of other people to overhear

Can you explain how the situation is during your visit to PHC?

Explore:

-How is the setting of the PHC and rooms?

-How does the setting affects your privacy?

How important is privacy during the PHC visit for you?

Explore:

Do you feel (un)comfortable with the current situation? Do you have any concerns? Do you think your privacy is sufficiently protected in PHC?

What do you think about your data privacy in PHC? (That is in medical records, computer, and online information systems)

**Non-PHC users**

Where do you usually go to seek medical care? What is the reason of choosing this particular healthcare facility?

What do you think about the healthcare services provided at PHC?

What/how was your experience there?

What do you think / What is your perception about privacy in PHC?

**Doctors, management and other PHC staff**

How does a usual working day look like for you in the PHC?

Can you explain the importance of privacy in PHC for you?

What is your experience with patient privacy (or privacy in general) in PHC?

Do you have any thoughts or concerns about the privacy situation in PHC?

What do you think about patient data privacy in PHC? (That is in medical records, computers, and online information systems)
